# Editor's note: new facial nerve section and new editorial board member

**DOI:** 10.1186/1746-160X-4-6

**Published:** 2008-03-11

**Authors:** Thomas Stamm

**Affiliations:** 1 Poliklinik für Kieferorthopädie, Universitätsklinikum Münster, Westfälische Wilhelms-Universität, Münster, Germany

## Editorial

As Editors of *Head & Face Medicine *we are pleased to announce the timely addition of a new subspecialty *Facial Nerve *to the scope of our multi-disciplinary journal. The Journal Development Committee has decided to create this new section, and the subspecialty's growing importance is reflected by the creation of a new editorial board position. Rainer Laskawi, MD, Professor of Otolaryngology at the ENT Department of the University of Göttingen, has agreed to accept this new editorial board position. We are grateful to Professor Laskawi, winner of the Ludwig Haymann Award of the German ENT Society, for his willingness to offer his outstanding expertise to work with us for the benefit of the journal and the scientific community.

Rainer Laskawi brings a wealth of experience to this new position. He experimentally studied the effects of peripheral nerve lesions (mainly facial nerve) on the central nervous system, especially on the supranuclear brain structures such as the motor cortex. His research group found fundamental short time changes in the functional cortical reorganization following different types of facial nerve lesions. Using glial cell markers, Laskawi and coworkers could demonstrate structural changes in later stages of cortical reorganization.

Rainer Laskawi's research interests focus on clinical problems associated with the facial nerve and the salivary glands, and he also studied the clinical use of botulinum toxin in many indications concerning the head, face and neck region. His research group first described the clinical use and effectiveness of botulinum toxin in sweat glands to treat focal hyperhidrosis, a research initiative that was greatly acknowledged by the German ENT Society. Jointly with Peter Roggenkämper (University of Bonn), he edited the volume *Botulinumtoxin – Therapie im Kopf-Hals-Bereich*.

Rainer Laskawi is President of the *Sir Charles Bell Society (SCBS)*, an international multi-disciplinary non-profit organization created at the *VII International Facial Nerve Symposium in Cologne (1992)*, dedicated to collection, dissemination and interchange of ideas relating to the facial nerve. As the President of the SCBS, member of the Scientific Board of the *German Dystonia Society*, and Section Editor of *Head & Face Medicine *he holds key positions for encouraging and disseminating multi-disciplinary thinking among students, clinicians, and researchers with a scientific interest in the facial nerve.

*Head & Face Medicine *is deeply grateful that Professor Laskawi will spend his valuable time to hold this new Section Editor position. His contribution will be important in helping shape the journal's course over the coming years.

**Figure 1:**
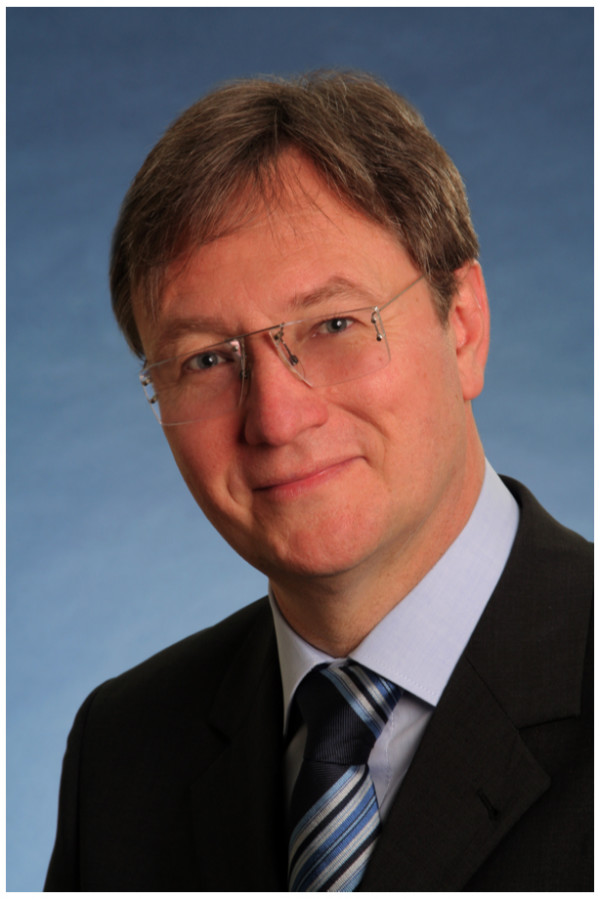
Prof. Dr. Rainer Laskawi.

